# P-179. Comparative Clinical And Therapeutic Features Between Rickettsia conorii And Rickettsia typhi Infections

**DOI:** 10.1093/ofid/ofaf695.403

**Published:** 2026-01-11

**Authors:** Fatma Hammami, Khaoula Rekik, Amal Chakroun, Makram Koubaa, Fatma Smaoui, Chakib Marrakchi, Mounir Ben Jemaa

**Affiliations:** Infectious Diseases Department, Hedi Chaker University Hospital, University of Sfax, Tunisia, Sfax, Sfax, Tunisia; Infectious Diseases Department, Hedi Chaker University Hospital, University of Sfax, Tunisia, Sfax, Sfax, Tunisia; Infectious Diseases Department, Hedi Chaker University Hospital, University of Sfax, Tunisia, Sfax, Sfax, Tunisia; Infectious Diseases Department, Hedi Chaker University Hospital, University of Sfax, Tunisia, Sfax, Sfax, Tunisia; Infectious Diseases Department, Hedi Chaker University Hospital, University of Sfax, Tunisia, Sfax, Sfax, Tunisia; Infectious Diseases Department, Hedi Chaker University Hospital, University of Sfax, Tunisia, Sfax, Sfax, Tunisia; Infectious Diseases Department, Hedi Chaker University Hospital, University of Sfax, Tunisia, Sfax, Sfax, Tunisia

## Abstract

**Background:**

*Rickettsia conorii* and *Rickettsia typhi*, although both belonging to the spotted fever group, present distinct clinical patterns that are crucial for early diagnosis and management. The aim of our work was to compare the clinical and therapeutic features between *Rickettsia conorii* and *Rickettsia typhi* infections.

**Methods:**

We conducted a retrospective study including all patients hospitalized in the infectious diseases department for rickettsiosis between 1995 and 2024. The diagnosis was confirmed by serological tests (seroconversion) or a positive polymerase chain reaction assays for *Rickettsia*.

**Results:**

We encountered 472 cases. There were 286 cases of *Rickettsia conorii* (60.6%) and 186 cases of *Rickettsia typhi* (39.4%). A male predominance was noted among *Rickettsia conorii* (54.2%) and *Rickettsia typhi* (54.8%) cases (p=0.89). Cephalalgia (75.5% vs 63.4%; p=0.005) and arthralgie (76.9% vs 68.3% ; p=0.038) were significantly more frequent among *Rickettsia conorii* cases. Fever (99.7% vs 100% ; p=1) and vomiting (44.8% vs 44.9% ; p=0.98) were reported among *Rickettsia conorii* and *Rickettsia typhi* cases. The occurence of inoculation eschar (29% vs 18.3%; p=0.008), maculopapular skin rash (83.6% vs 74.6% ; p=0.017) and retinitis (12.7% vs 2%; p=0.045) was significantly higher among *Rickettsia conorii* cases. Severe forms of rickettsiosis were significantly more frequent with *Rickettsia conorii* (23.4% vs 15.7% ; p=0.041). Hepatic cytolysis (66.8% vs 69.4% ; p=0.55), leucopenia (16.4% vs 13.4% ; p=0.37) and thrombocytopenia (58.5% vs 57,8% ; p=0.88) were noted among *Rickettsia conorii* and *Rickettsia typhi* cases. Bitherapy was more frequently prescribed for cases of *Rickettsia conorii* (9% vs 4% ; p=0.046). Doxycycline was prescribed for *Rickettsia conorii* (64.7%) and *Rickettsia typhi* (71.5%) cases (p=0.12). Ciprofloxacine was prescribed for *Rickettsia conorii* (15.7%) and *Rickettsia typhi* (12.9%) cases (p=0.39).

**Conclusion:**

*Rickettsia conorii*, the predominant species, was associated with severe forms of rickettsiosis. Cephalalgia, arthralgia, inoculation eschar, rash, and retinitis were significantly more common among *Rickettsia conorii* cases, in comparison with *Rickettsia typhi*. Bitherapy was more frequently prescribed for *Rickettsia conorii* cases.

**Disclosures:**

All Authors: No reported disclosures

